# Higher genome variability within metabolism genes associates with recurrent *Clostridium difficile* infection

**DOI:** 10.1186/s12866-021-02090-9

**Published:** 2021-01-28

**Authors:** Maria Kulecka, Edyta Waker, Filip Ambrozkiewicz, Agnieszka Paziewska, Karolina Skubisz, Patrycja Cybula, Łukasz Targoński, Michał Mikula, Jan Walewski, Jerzy Ostrowski

**Affiliations:** 1 Department of Genetics, Maria Sklodowska-Curie National Research Institute of Oncology, Roentgena 5, 02-781 Warsaw, Poland; 2 Department of Gastroenterology, Hepatology and Clinical Oncology, Centre of Postgraduate Medical Education, 02-781, Warsaw, Poland; 3 Department of Clinical Microbiology, Maria Sklodowska-Curie National Research Institute of Oncology, 02-781 Warsaw, Poland; 4 Department of Lymphoproliferative Diseases, Maria Sklodowska-Curie National Research Institute of Oncology, 02-781 Warsaw, Poland

**Keywords:** *Clostridium difficile*, Infection, Recurrence, Whole genome sequencing, Prophage

## Abstract

**Background:**

*Clostridium difficile (C. difficile)* is a major source of healthcare-associated infection with a high risk of recurrence, attributable to many factors such as usage of antibiotics, older age and immunocompromised status of the patients. *C. difficile* has also a highly diverse genome, which may contribute to its high virulence. Herein we examined whether the genome conservation, measured as non-synonymous to synonymous mutations ratio (dN/dS) in core genes, presence of single genes, plasmids and prophages increased the risk of reinfection in a subset of 134 *C. difficile* isolates from our previous study in a singly hemato-oncology ward.

**Methods:**

*C. difficile* isolates were subjected to whole-genome sequencing (WGS) on Ion Torrent PGM sequencer. Genomes were assembled with MIRA5 and annotated with prokka and VRprofile. Logistic regression was used to asses the relationship between single gene presence and the odds of infection recurrence. DN/dS ratios were computed with codeml. Functional annotation was conducted with eggNOG-Mapper.

**Results:**

We have found that the presence of certain genes, associated with carbon metabolism and oxidative phosphorylation, increased the odds of infection recurrence. More core genes were under positive selective pressure in recurrent disease isolates – they were mostly associated with the metabolism of aminoacids. Finally, prophage elements were more prevalent in single infection isolates and plasmids did not influence the odds of recurrence.

**Conclusions:**

Our findings suggest higher genetic plasticity in isolates causing recurrent infection, associated mainly with metabolism. On the other hand, the presence of prophages seems to reduce the isolates’ virulence.

**Supplementary Information:**

The online version contains supplementary material available at 10.1186/s12866-021-02090-9.

## Background

*Clostridium difficile* (reclassified in 2016 into a new *Clostridiodes* genus, along with *Clostridium mangenotii*, with which it shares a 94.7% similarity within 16 s rRNA gene [1]) is an anaerobic, spore-forming, Gram-positive bacterium, prevalent in the environment, as well as human gastrointestinal tract: it is mainly present in infants [2, 3], in whom the asymptomatic colonization occurs. On the other hand, in adults, *C. difficile* colonization is characterized by life-threatening infection, with symptoms ranging from moderate diarrhea to severe colitis and/or megacolon [4, 5]. In Europe, the majority (74.6%) of *C. difficile* cases are healthcare-associated (HA). The mean incidence per 10,000 patient-days is at 2.38, but some countries, such as Estonia, Lithuania and Poland present with much higher numbers [6]. In large European hospital surveys from 10% [7] to 16% [6] *Clostridium difficile* infection (CDI) cases are associated with major complications, requiring admission to the intensive care unit and resulting in the death rate of 7 and 4% respectively.

Another major concern when dealing with CDI is its tendency of recurrence which, according to the European Society of Clinical Microbiology and Infectious Diseases, is defined as a relapse of CDI clinical symptoms within 2–8 weeks of successful treatment of the initial episode. The recurrent CDI may be due to a relapse of the previous CDI by the same strain or reinfection by a different strain [8–10]. While distinguishing of recurrence due to relapse or from recurrence due to reinfection is not feasible in daily practice, the method of choice in this distinction is bacterial genotyping [9]. Reported recurrence rates vary between 5 and 50%, but most of them are between 10 and 20% [11]. Various recurrence risk factors have been identified, including continued use of antibiotics not associated with CDI treatment [12], particularly cephalosporins [13, 14], older age [12, 15], HA diseases [15, 16], length of hospitalization [15] and usage of gastric acid suppressors such as proton pump inhibitors [10, 12, 17, 18]. Immunocompromised patients also typically present a higher risk of infection recurrence [19, 20].

In addition, the high plasticity of *C. difficile* genome may also account for its virulence and notorious recurrence. Sequenced *C. difficile* genomes’ size typically ranges from 4.1 to 4.3 Mb [21–24] and is much larger not only than most of the related species but also most of the *Firmicutes* phylum. A large genome can be a sign of exceptional adaptability to various conditions, often for prolonged periods [25]. Indeed, a large number of differentially expressed genes during infection were found to be associated with adaptation mechanisms such as stress response and sporulation [26]. The *C. difficile* 630 genome was also found to contain an unusually high proportion (11%) of mobile elements, including transposons and prophages [27]. The horizontal gene transfer, occurring through these elements is particularly important in the acquisition of resistance to various antimicrobial agents [24]. Finally, the *C. difficile* genome can be altered through point mutations and inversions: in the comparison between 3 strains: 630, R20291 and CD196 39 variations such as these were found [23, 28].

In this context, the aim of our work was twofold. Firstly, to investigate and establish the core genome and pangenome of Clostridial species recovered from patients with single and multiple CDIs hospitalized in a single hemato-oncology ward through a period of ten years, both on genetic and functional level. Secondly, to investigate the dN/dS ratio in the core genome. This data was used to compare strains, which cause recurring infections to those, which affected the patient only once.

## Results

### *Clostridium difficile* core genome

**Fig. 1:**
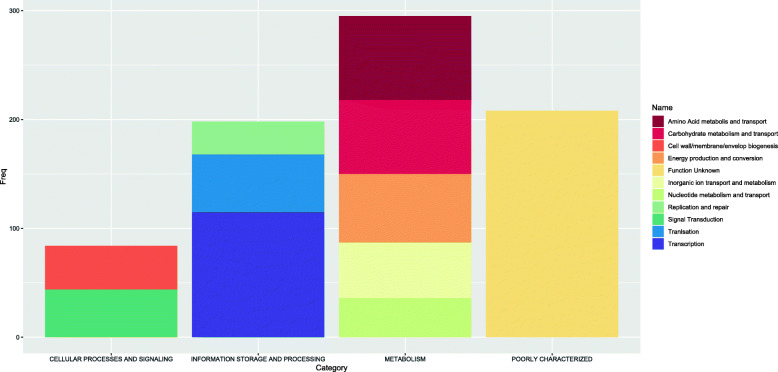
Main core gene categories as characterized by COG classification

**Fig. 2:**
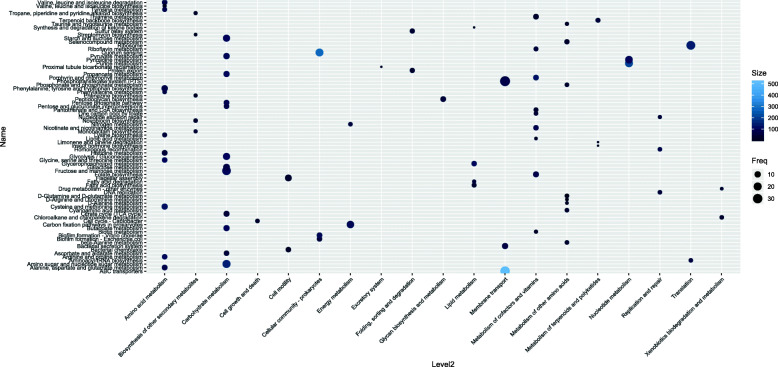
Main pathways present in *Clostridium difficile common* core genome. The pathways represented here had at least 0.1 ratio between number of genes and size of KEGG pathway (according to number of KEGG orthologies)

There were 965 core genes discovered (i.e. present in more than 90% of samples) shared between isolates from ST1 and ST42. Apart from 208 proteins with poorly characterized function, the most abundant COG categories concerned metabolism (including carbohydrate and amino acid metabolism), information storage and processing (transcription and translation) and cellular processes and signaling (signal transduction and cell wall biogenesis) (Fig. 1, Supplementary Table 1). 135 KEGG Pathways (on a third level of classification) were represented in the common core genome (Supplementary Table 2). The most represented pathways (i.e. the ones with the highest ratio of present genes to the total number of KEGG orthologies) are associated with membrane transport, aminoacid, carbohydrate and lipid metabolism, bacterial cell motility, genetic information processing, energy metabolism and metabolism of cofactors and vitamins (Fig. 2). 62% pathways present within the core genome are associated with metabolism.

### Specific gene presence as a predictor of recurrence

In the logistic regression model, there were 5264 genes tested; while 192 reached statistical significance at a nominal *p*-value of 0.05, none of them were significant after correction for multiple hypothesis testing with the FDR method (Supplementary Table 3). 7 pathways are enriched in gene set which gives higher odds of infection recurrence, however, in only one of them the enrichment score reaches the highest value at rank smaller than 192: Oxidative phosphorylation (Table 1, Supplementary Table 4).

**Table 1:** Gene set enrichment analysis for genes ranked according to odds of infection recurrence. Size – total size of pathway in tested dataset, ES – enrichment score, NES – normalized enrichment score, pvalue – *p*-value in GSEA analysis (permutation test), padjust – pvalue after adjustment for multiple testing, rank – rank with peak enrichment score, core enrichment – gene names (if available) present in core enrichment. Adjusted *p*-values < 0.05 were considered significant

ID	Description	setSize	ES	NES	pvalue	padjust	rank	core enrichment
ko00190	Oxidative phosphorylation	15	0.87	2.12	2.19E-04	1.22E-02	160	*ntpG/ntpI/atpD/ntpB/ppaC/hydA*
ko01200	Carbon metabolism	67	0.50	1.71	9.59E-04	2.07E-02	899	*ato1/acoB/cooS1/rpiB/fdhF/serA/ppaC/acoC/fwdE/pgk/glcK/fba2/pyc/bcd_1/tal/tklB/pfkB/pgi/dpaL/fbp/nadB/rpe/dpaL/nifJ/gap*
ko01120	Microbial metabolism in diverse environments	199	0.37	1.47	1.11E-03	2.07E-02	1245	*group_10292/ato1/mngA/acoB/licR/cooS1/rpiB/fdhF/serA/group_3100/group_15830/ppaC/argD/group_10260/acoC/fwdE/group_18370/group_14018/mtlA/pyrK/group_14004/group_20309/pgk/hemB/glcK/fba2/pyc/cobA/leuC/group_7776/bcd_1/group_1934/dfa1/group_12907/group_4079/tal/group_11132/tklB/lysC/rnhA/pfkB/group_11374/pgi/group_7372/group_11044/fbp/nadB/group_607/asrA/asrB/MA20_09190/rpe/group_12912/thrC/group_1435/group_1680/nifJ/gap/hom/rnhA/adhE/xdhA1/fucA/acsD/group_16780/group_14000/asrC/group_7768/icd/ytjP/sucD/group_16251/group_8676/rpiB/gutB/algC/gatB/hom/rpe/cooS1/group_18922/pmmB/group_18142*
ko03010	Ribosome	25	0.66	1.82	1.58E-03	2.20E-02	636	*rpsK/rpsS/rpsE/rpsL/rplO/rplB/rplA/rpmH/rpmD*
ko01130	Biosynthesis of antibiotics	118	0.41	1.53	3.07E-03	3.10E-02	922	*dxs/ato-1/acoB/ispD/rpiB/serA/argD/group_10260/lysN/acoC/purH/aspC/pgk/ilvC/glcK/fba2/argJ/group_7776/galU/tal/tklB/lysC/pabC/pfkB/group_11374/ilvB/pgi/group_7372/dpaL/glmS/argB/fbp/nadB/argC/rpe/dpaL/nifJ/gap/hom/group_15453*
ko01210	2-Oxocarboxylic acid metabolism	19	0.68	1.77	3.33E-03	3.10E-02	799	*argD/lysN/aspC/ilvC/argJ/leuC/lysC/pabC/ilvB/argB/argC*
ko00710	Carbon fixation in photosynthetic organisms	11	0.78	1.75	4.75E-03	3.79E-02	1223	*rpiB/pgk/fba2/tklB/fbp/rpe/gap/rpiB/rpe*

### Gene conservation differences between recurrent and single infection

515 core genes were included in this analysis. Sequences were analyzed separately for recurrent and one-time infections. For recurrent infections, 65 genes had sites under positive selection, while for single infections this number was at 17. 25 genes under positive selection only in recurrent sequences could be functionally annotated with KEGG pathways. Most of them are associated with metabolism, mainly of aminoacids and secondary metabolites. Other genes of interest include toxin B and *cheC,* involved in bacterial chemotaxis (Table 2, Supplementary Table 5).

**Table 2:** Genes with functional annotation, with sites under positive selection pressure in recurrent but not in single infections. Pvalues are given for log-likelihood ratio test between M1a and M2a models with or without adjustment for multiple hypothesis testing. Adjusted *p-*values < 0.05 were considered significant

gene	*p*value-recurrent	*p*value-single	padjust-recurrent	padjust-single
*tyrB*	2.12E-05	9.97E-01	3.21E-04	1.00E+ 00
*cdd3*	6.02E-12	1.06E-01	1.63E-10	1.00E+ 00
*fatC*	1.10E-04	7.96E-01	1.45E-03	1.00E+ 00
*opuCC*	1.41E-03	6.55E-01	1.46E-02	1.00E+ 00
*asnB*	2.22E-07	8.77E-02	4.23E-06	1.00E+ 00
*glsA*	1.81E-07	9.70E-01	3.59E-06	1.00E+ 00
*gltA*	8.87E-05	9.96E-01	1.23E-03	1.00E+ 00
*group_18063*	1.90E-03	9.97E-01	1.77E-02	1.00E+ 00
*selA*	1.71E-03	5.32E-02	1.66E-02	1.00E+ 00
*prdF*	0.00E+ 00	9.60E-01	0.00E+ 00	1.00E+ 00
*rapL*	1.26E-07	8.07E-01	2.66E-06	1.00E+ 00
*cheC*	2.29E-03	7.83E-01	2.11E-02	1.00E+ 00
*mtnN*	1.89E-05	7.46E-01	2.95E-04	1.00E+ 00
*accA*	5.01E-03	6.96E-01	4.09E-02	1.00E+ 00
*gph*	2.73E-03	5.43E-01	2.43E-02	1.00E+ 00
*hpt*	3.15E-03	8.94E-01	2.70E-02	1.00E+ 00
*hydE*	1.06E-04	9.99E-01	1.44E-03	1.00E+ 00
*actI*	1.14E-03	9.16E-01	1.25E-02	1.00E+ 00
*folC*	0.00E+ 00	9.83E-01	0.00E+ 00	1.00E+ 00
*xylA*	4.29E-15	9.78E-01	1.30E-13	1.00E+ 00
*csdA*	1.63E-171	9.09E-01	1.05E-169	1.00E+ 00
*dacF*	1.62E-09	4.24E-02	3.89E-08	8.73E-01
*tcdB*	7.06E-20	1.31E-02	3.03E-18	3.98E-01
*rnr*	7.34E-16	8.11E-01	2.52E-14	1.00E+ 00
*regB*	2.47E-07	6.61E-03	4.54E-06	2.43E-01

### Mobile genetic elements

Three different plasmids were discovered in our strains: pCD6, pCDBI1 and DSM 1296. Plasmids were present in 5% (5 out of 98) strains which cause recurrent infection and in 11% strains which did not (4 out of 36). This difference is not statistically significant (*p*-value 0.25, Fisher’s exact test, Table 3).

**Table 3:** Types of plasmids discovered is sequenced strains

Plasmid ID	Copy number	SampleID	Type of infection
pCD6	4.06	3364M15	Recurrent
DSM 1296	3.14	712M15	Recurrent
pCD6	6.18	500M12	Single
pCD6	4.43	3309M13	Recurrent
pCD6	3.39	178938	Single
pCD6	4.29	3544M15	Recurrent
pCDBI1	2.84	925M12	Single
DSM 1296	2.79	925M12	Single
pCD6	5.67	2401M11	Recurrent
pCDBI1	2.6	561M17	Single

Eleven prophage elements differed in frequency between single and recurrent infections at a nominal *p-*value < 0.05, however, none of them remained significant after multiple hypothesis testing correction. Most of them originated from phages CD119 and C2 (Table 4, Supplementary Table 6).

**Table 4:** Prophage sequences present in sequenced clostridial genomes. *P*value – *p-*value in Fisher’s exact test, padjust – *p-*value, adjusted for multiple hypothesis testing, odds ratio – odds of recurrent infection, %single/recurrent – percentage of sequences with prophage present in single/recurrent infections. Adjusted *p*-values < 0.05 were considered significant

	pvalue	padjust	odds ratio	%single	%recurrent
Prophage_134287341 NC_009231 putative scaffold protein {Clostridium phage phiC2}	4.85E-03	1.60E-01	0.11	17%	2%
Prophage_90592671 NC_007917 putative lysin {Clostridium phage phi CD119}	6.91E-03	1.60E-01	0.26	31%	10%
Prophage_90592670 NC_007917 putative holin {Clostridium phage phi CD119}	1.50E-02	1.60E-01	0.32	81%	57%
Prophage_80159693 NC_007581 putative IS transposase (OrfA) {Clostridium phage c-st}	1.64E-02	1.60E-01	0.23	19%	5%
Prophage_90592681 NC_007917 repR RepR putative repressor {Clostridium phage phi CD119}	1.64E-02	1.60E-01	0.23	19%	5%
Prophage_90592642 NC_007917 putative head protein {Clostridium phage phi CD119}	1.84E-02	1.60E-01	0.08	11%	1%
Prophage_134287355 NC_009231 putative tail tape measure protein {Clostridium phage phiC2}	3.02E-02	2.07E-01	0.33	28%	11%
Prophage_134287357 NC_009231 putative hydrolase {Clostridium phage phiC2}	3.24E-02	2.07E-01	0.20	14%	3%
Prophage_134287371 NC_009231 putative amidase/endolysin {Clostridium phage phiC2}	3.60E-02	2.07E-01	0.31	22%	8%
Prophage_134287356 NC_009231 putative LysM {Clostridium phage phiC2}	4.20E-02	2.07E-01	0.27	19%	6%
Prophage_90592656 NC_007917 xkdP XkdP protein {Clostridium phage phi CD119}	4.37E-02	2.07E-01	0.40	78%	58%

## Discussion

The *C. difficile* genome is subject to constant changes, as estimated, it acquires between 1 and 2 mutations per genome per year [25, 29]. However, not all mutations are viable, and some clones may become subject to purifying selection. The selective pressure can be described with the dN/dS ratio, i.e. the ratio of non-synonymous to synonymous mutations. The dN/dS ratio significantly smaller than 1 suggests strong purifying selection, while in most *C. difficile* genomes the reverse situation is observed where the dN/dS is actually higher than 1 [24, 30]. This suggests less efficient purging of novel mutations, possibly contributing to *C. difficile* high genetic diversity and adaptability. With such plasticity and diversity, it is difficult to establish the exact size of *C. difficile* core genome and pangenome. Usually, the orders of magnitude of about 1000 genes are given [31, 32], but some researchers give figures as high as 3000 [33], which would be the most of *C. difficile* genome. Nevertheless, the estimated size of the core genome usually varies between 16% [34] and 24% [31], which is much lower than most bacterial species. For instance, in pathogenic *Streptococcus agalactiae*, the core genome constitutes about 80% of the whole genome, in *Helicobacter pylori* 77 and 46% in *Streptococcus pneumoniae* [35]. On the other hand, the size of the core and essential genome was estimated to be composed of 404 genes [36], a number comparable to other bacterial species, such as *Pseudomonas aeruginosa* (321 genes [37]) and *Yersinia pestis* (about 500 genes [38]).

The *C. difficile* core genome is usually estimated to comprise about 1000 genes [31], involved mainly in pathways related to metabolism (of aminoacids and carbohydrates), genetic information processing, cell motility and signal transduction [31, 34], the unsurprising functions in the core genome. Additionally, many clostridial core genes are associated with virulence, including toxins, cell surface proteins, flagellar proteins and antibiotic resistance factors [34]. Our study is in line with previous findings, with the core genome of 965 genes, present in the most prevalent strains. Apart from typical house-keeping pathways, we have identified several KEGG pathways associated with virulence such as beta-Lactam and vancomycin resistance, biofilm formation and flagellar assembly.

It is believed that highly adaptive metabolism is one of the key contributors to *C. difficile* virulence. *C. difficile* has two main energy sources: aminoacids and sugars. Some aminoacids (such as leucine, valine, proline) contribute to ATP formation via the so-called Stickland pathway [39] while other aminoacids (including cysteine, threonine, serine) and sugars contribute to energy production via central carbon metabolism and TCA cycle [40]. Furthermore, *C. difficile* exhibits some autotrophic characteristics, including genes from the Wood-Ljungdahl pathway in 4 sequenced genomes that allow an autotrophic growth by generating energy from CO_2_ and H_2_ via this pathway [41]. Production of toxin A and B is also correlated with alterations in central carbon metabolism with fluxes changing from butanoate to lactate synthesis [42]. In our work, genes whose presence increased the odds the reinfection are mainly associated with metabolism and energy production: Oxidative phosphorylation, Carbon metabolism, 2-Oxocarboxylic acid metabolism and Carbon fixation in photosynthetic organisms. This may suggest that infection recurrence is associated with altered metabolism and alternative means of energy production rather than the presence of additional virulence factors. However, the practical significance of this discovery in the aspect of new antimicrobial targets for *C. difficile* remains to be uncovered. Historically, bacterial central metabolism (including carbon metabolism) has not been reported as a potential source of new antimicrobial targets, since it has been believed that homology between crucial microbial and human enzymes is simply too high [43]. On the other hand, recent studies identified potential antimicrobial drug targets within carbon metabolism and fixation pathways in MRSA [44] and *Mycobacterium* *tuberculosis* [45]. While the aforementioned works are based on *in silico* methods, their practical utility remains to be proven.

Virulence-conferring plasmids are common in enteropathogenic bacteria, such as *Shigella* spp [46] and *Escherichia coli* [47]. They contribute to bacterial virulence by carrying genes associated with resistance against antimicrobial agents (such as plasmids R100 in *Shigella flexneri 2b* which contributes to resistance to sulfonamides, chloramphenicol, tetracyclines and streptomycin [48]) as well as host cell adhesion and invasion (plasmid pO157 in *E. coli* [49]). Two main plasmids were described in *C.difficile:* pCD6 [50] and pCD630 [27] – both are relatively small (less than 10 kb) in comparison with virulence-conferring plasmids, such as pO157, which is 93.6 kb in size [51]. Recently, plasmids larger than 40 kb were discovered [52]. However, while historically plasmid-typing was found to be useful in tracing and typing nosocomial CDI [53, 54], no clear associations between virulence and plasmid presence could be drawn for *C. difficl*e [52, 55]. Only recently a plasmid pMETRO, conferring resistance to metronidazole has been discovered [56]. In our study, plasmids were discovered in 6.7% isolates. None of the isolates were resistant to metronidazole and unsurprisingly pMETRO was not discovered in any of them. There was no statistically significant difference between plasmid fraction detected in one-time and recurrent infection isolates. Therefore, we believe our study reinforces the hypothesis that plasmids contribute little to *C. difficile* virulence and recurrence.

Prophages contribute to the evolution and virulence of most bacterial pathogens, including virulence and recurrence of *C. difficile* [57–59]. Prophages are abundant within *C. difficile* genome – up to 2018, at least 26 mobile element sequences were described [60]. They manifest a large variety of functions in *C. difficile*: while CD119 represses expression of five clostridial pathogenicity locus (PaLoc) genes [61], other prophages may promote virulence: upon infection with CD38–2, up to two-fold rise in toxin A and B was detected in hypervirulent BI/NAP1/027 (ST1) strain [58]. Another prophage, Semix9P1, was determined to carry a fully functional binary toxin [62]. In our study, prophage fragments were discovered, coming from two phages: CD119 and C2. Phage CD119 contains a gene, encoding RepR protein, which downregulates the expression of toxins A and B indirectly controlling the expression of tcdR, the toxin gene regulator [61]. *RepR* gene was found in almost 20% of single infection isolates and only in 5% recurrent infection isolates. This may suggest weakened virulence due to the prophage infection at least in some of the single infection isolates. On the other hand, phage C2 infection was found to affect the measured levels of toxin B in *C. difficile* isolates through the production of holins, proteins that disrupt the membrane and increase bacterial secretion [63]. However, after correction for multiple hypothesis testing none of 11 prophage elements uncovered in this study, including C2 phage originated holins, differed in frequency between single and recurrent infections.

Finally, high adaptability and increased virulence may be attributed to the beneficial point mutations which do not become subject to purifying selection. While it is expected for the core genome to be highly conserved, some of the clostridial core genes were found to be under positive selection. For instance, He et al. [24] identified 12 such sequences, including membrane proteins and response regulators. The dN/dS ratios for core genomes were higher than 1 for both strains analyzed by Murillo et al. [30] Recently, Kumar et al. [32] have proposed that *C. difficile* is undergoing active speciation and characterized genes under positive selective pressure which were associated with sporulation and sugars' metabolism. In our study, more genes were found to be under positive selective pressure in recurrent infection isolates that in single ones. While this may point to higher genetic adaptability of recurrent isolates, in this case other explanations should be also considered. First of all, we had a larger number of sequences from recurrent isolates, which increases probability of detecting positive selection. In addition, in closely related lineages the dN/dS ratios were proved to be higher since the purifying selection did not have time to purge mutations [64]. Nevertheless, genes under positive selection in recurrent infections in our study seem to share some of the characteristics with those designated by He et al. and Kumar et al. [24, 32]. In line with He et al., we have ABC transporters (*cdd3, fatC* and *opuCC*) and two-component system members (*regB*) with sites under positive selection. We have also observed changes in metabolism, but they concern aminoacids rather than sugars, similar to Kumar et al. study. Interestingly, toxin B also has been found to be under positive selective pressure in recurrent isolates. Toxin B was found to be crucial in *C. difficile* virulence [65], with toxinB(+) toxinA (−) mutants being fully virulent, while in the reverse situation the virulence is attenuated [66]. Genetic variability within toxin sequences is a well-known phenomenon [67], with 34 toxinotypes currently defined [68]. While our results may suggest an existing selective pressure on toxin B gene, it is also worth noting that most of the toxinotypes are known since the beginning of research on the subject and on a larger scale prevalence of alternative toxinotypes is attributable to local outbreaks [67].

## Conclusions

To conclude, we have managed to thoroughly analyze how genetic mobility influences infection recurrence in CDI. We have confirmed the lack of significance of plasmid-related virulence, as well as reinforced the role of prophages in the virulence-related mechanisms. This seems to be of particular importance since phage therapy seems like a beneficial alternative due to limited antibiotics available for the treatment of CDI [69]. We have also observed changes in metabolism-related genes, both in prevalence (shell genes), as well as in conservation (core genes).

## Methods

Research involving human data was performed in accordance with the Declaration of Helsinki.

The study was approved by the Maria Skłodowska-Curie National Research Institute of Oncology Ethics Committee (number 40/2018). In line with the opinion of the Bioethics Committee at Maria Skłodowska-Curie National Research Institute of Oncology our study did not require informed consent for the following reasons: This is a retrospective study describing the genetic differences between *C. difficile* strains but not between patients; bacterial strains were isolated during routine diagnostics and then banked over the course of one to 10 years; most of these patients are already dead.

As reported recently [70], between 2008 and 2018, all patients hospitalized at the Department of Lymphoma with healthcare-associated diarrhea (defined as ≥3 stools within a 24-h period arising over the third day after hospital admission) underwent testing at the Department of Clinical Microbiology to detect pathogenic *C. difficile* toxins A and B. Tests were performed using the *C. difficile* TOX A/B kit (TechLab). Subcultured single colonies from 134 available culture-positive isolates were subjected to whole-genome sequencing (WGS) on Ion Torrent PGM sequencer. Of these, 36 isolates were recovered from patients (18 women and 18 men) with a single CDI, and 98 were recovered from 44 patients (19 women and 25 men) with multiple CDIs. Multi-locus sequence typing results are taken from this publication as well.

### Genome assembly and annotation

The sequenced genomes were assembled with MIRA 5 (https://sourceforge.net/projects/mira-assembler/) genome assembler [71], using parameters set specific for Ion Torrent sequencing technology. The assembled sequences were annotated with prokka [72] version 1.13 (https://github.com/tseemann/prokka), using a minimal contig length of 1000, proteins from RT027 CD196 strain as a list of trusted proteins and a “metagenome” option to improve annotation in case of large genome fragmentation. The pan-genome calculation was conducted with roary [73] version 3.12 (https://sanger-pathogens.github.io/Roary/).

### Functional annotation of coding sequences and mobile genetic elements

The functional annotation of identified CDS was conducted with eggNOG-Mapper [74] version 2.0 (https://github.com/eggnogdb/eggnog-mapper), using eggNOG [75] categories as well as KEGG [76] pathways. All visualizations were performed with R package ggplot2 [77]. Mobile elements, specifically the prophages were annotated with VRProfile [78] (https://tool-mml.sjtu.edu.cn/STEP/STEP_VR.html). Plasmids were identified with PlasmidSeeker [79] (https://github.com/bioinfo-ut/PlasmidSeeker).

### Gene presence as a predictor of disease recurrence

The influence of a single gene on odds of disease recurrence was assessed with a logistic regression model, with ST as a confounding variable. The analysis was conducted only for genes present in more than 15% and less than 90% of cases. The *p*-values from this model on a PHRED scale (i.e. transformed with negative logarithm with base 10) served as metric for GSEA (Gene Set Enrichment Analysis) conducted with function from Cluster Profiler [80] package, with 10,000 permutations and maximum gene set size of 200. The metric was negative for genes which decreased the odds of recurrence.

### Gene conservation in recurrent and one-time infections

In order to compute gene conservation, the CDSs of core genes from STs 1 and 42 were translated into proteins with translate function from BioStrings R package [81] version 2.46 and option to resolve ambiguous codons. The sequences were then aligned with msa function from R package msa [82] version 1.14 (using default parameters and default aligner ClustalW [83]). The protein alignment was then converted to codon alignment with pal2nal script [84]. The presence of genetic recombination was verified with PhiPack [85] software and analysis was not continued only if the sequences passed 2 tests present in the package. The dN/dS ratios among different sites were then assessed with codeml (part of PAML4 [86] package - http://abacus.gene.ucl.ac.uk/software/paml.html), using a comparison of two models - nearly neutral (designed M1a in PAML manual - http://abacus.gene.ucl.ac.uk/software/pamlDOC.pdf) and positive selection (M2a). *P-*values were adjusted for multiple testing with Benjamini-Hochberg FDR correction [87].

## Supplementary Information

**Additional file 1 **Further data are available as Supplementary Tables: **Table S1.** COG categories prevalence in *C. difficile* core genome. **Table S2.** KEGG pathways present in core genome. **Table S3.** Logistic regression results for odds of infection recurrence after adjustment for ST. **Table S4.** Gene set enrichment analysis for results of logistic regression. **Table S5.** Log-likelihood ratio test results for comparison between M2a and M1a models. **Table S6.** Fisher’s exact test results for prevalence difference in prophage sequence between single and recurrent infections.

## Data Availability

The datasets generated for this study can be found as raw fastq files in Sequence Read Archive with accession number PRJNA608241 (https://www.ncbi.nlm.nih.gov/bioproject/PRJNA608241/).
